# Plasma Pentraxin3 is a Novel Marker for Nonalcoholic Steatohepatitis (NASH)

**DOI:** 10.1186/1471-230X-8-53

**Published:** 2008-11-14

**Authors:** Masato Yoneda, Takashi Uchiyama, Shingo Kato, Hiroki Endo, Koji Fujita, Kyoko Yoneda, Hironori Mawatari, Hiroshi Iida, Hirokazu Takahashi, Hiroyuki Kirikoshi, Masahiko Inamori, Yuichi Nozaki, Noritoshi Kobayashi, Kensuke Kubota, Satoru Saito, Shiro Maeyama, Mina Sagara, Hiroyuki Aburatani, Tatsuhiko Kodama, Atsushi Nakajima

**Affiliations:** 1 Division of Gastroenterology, Yokohama City University Graduate School of Medicine, 3-9 Fuku-ura, Yokohama, Japan; 2 Kitakashiwa Rehabilitation Hospital, 265 Kashiwashita, Kashiwa, Japan; 3 Perseus Proteomics Inc., 4-7-6 Komaba, Meguro-ku, Tokyo, Japan; 4 Geneme Science Division, Research Center for Advanced Science and Technology, Graduate School of Medicine, The University of Tokyo, 4-6-1 Komaba, Meguro-ku, Tokyo, Japan; 5 Laboratory for Systems Biology and Medicine, Research Center for Advanced Science and Technology, Graduate School of Medicine, The University of Tokyo, 4-6-1 Komaba, Meguro-ku, Tokyo, Japan

## Abstract

**Background:**

The changes in the liver in nonalcoholic fatty liver disease (NAFLD) range over a wide spectrum, extending from steatosis to steatohepatitis (NASH). However it has remained difficult to differentiate between NASH and non-progressive NAFLD on the basis of the clinical findings alone.

**Aims:**

In this study we investigated the clinical usefulness of plasma Pentraxin3 (PTX3) levels to predict NASH. Plasma PTX3 was measured in 70 patients with histologically verified NAFLD (28 with non-NASH and 42 with NASH) and 10 healthy control subjects.

**Results:**

The plasma PTX3 level was significantly higher in the NASH cases than in the non-NASH cases (p = 0.0021) and control subjects (p = 0.045). And the plasma PTX3 level was significantly higher in the stages 3–4 NAFLD cases than in the stages 0–2 NAFLD cases (p < 0.0001). The PTX3 values were closely correlated with the stages of liver fibrosis (p < 0.0001, Kruskal-Wallis test). To detect NASH compared with non-NASH, the area under the curve for plasma PTX3 were 0.755, and to detect stages 3–4 NAFLD compared with stages 0–2 NAFLD, the area under the curve for plasma PTX3 were 0.850.

**Conclusion:**

This is the first study to demonstrate consistent and profound elevation of plasma PTX3 levels in NASH in comparison with non-NASH. The results suggest that plasma PTX3 levels may not only be laboratory values that differentiate NASH from non-NASH, but marker of the severity of hepatic fibrosis in NASH.

## Background

Nonalcoholic fatty liver disease (NAFLD) is one of the most common causes of chronic liver injury in many countries around the world [1,2]. It represents a spectrum of conditions that are histologically characterized by macrovesicular hepatic steatosis, and the diagnosis is made in patients who have not consumed alcohol in amounts sufficient to be considered harmful to the liver. The histological changes range over a wide spectrum, extending from simple steatosis, which is generally non-progressive, to nonalcoholic steatohepatitis (NASH), liver cirrhosis, liver failure, and sometimes even hepatocellular carcinoma [3,4]. Liver biopsy is recommended as the gold standard for both the diagnosis and staging of fibrosis in NASH patients [1,4,5], but it is invasive [6] and avoidance of liver biopsy would be desirable. Several clinical studies have attempted to identify serum markers that correlate with the severity of liver fibrosis in NASH patients. Many clinical variables have been proposed as predictors of severe fibrosis in NAFLD patients, including old age, underlying type 2 diabetes mellitus, obesity, serum transaminase level, platelet count, etc [7-9].

We have previously reported that measurement of the serum high-sensitivity C-reactive protein (CRP) level is clinically useful for the diagnosis of NASH [10]. CRP and serum amyloid P component (SAP) are well known members of the pentraxin family of proteins, which is subdivided into two subclasses according the length and structure of the molecules: long and short. The classical short PTXs, CRP and serum amyloid P, are acute-phase proteins in humans [11], that are produced in the liver in response to inflammatory mediators, most prominently IL-6. Long PTXs are characterized by an unrelated N-terminal domain coupled to a PTX-like C-terminal domain [12,13]. The prototypic long PTX3 is rapidly produced in response to Toll-like receptor engagement, tumor necrosis factor-α, and IL-1β and released by diverse cell types, including monocytes/macrophages, endothelial cells, vascular smooth muscle cells, fibroblasts, and adipocytes [14-19]. Plasma PTX3 levels have recently been found to be elevated in patients with vasculitis [20], acute myocardial infarction [21,22], and systemic inflammation or sepsis [23], however, there is no information about changes in PTX3 levels in NAFLD or NASH patients. We hypothesized that plasma PTX3 levels increase in patients with NASH, and investigated the clinical usefulness for the diagnosis and staging of liver fibrosis in NASH patients.

## Methods

### Patients

70 Japanese NAFLD patients (42 NASH and 28 non-NASH) and 10 healthy control subjects were recruited. All control subjects were confirmed to have normal liver function and no viral hepatitis infection or alcoholics. All of the 70 NAFLD patients were performed liver biopsy. The study was conducted with the approval of the Ethics Committee of Yokohama City University.

A detailed history was obtained and a physical examination performed on all the 70 NAFLD patients. The histological criteria for the diagnosis of NAFLD are the presence of macrovesicular fatty change in hepatocytes with displacement of the nucleus to the edge of the cell [24]. The criteria for exclusion from participation in the study: history of hepatic disease, such as chronic hepatitis C or concurrent active hepatitis B (serum positive for hepatitis B surface antigen), autoimmune hepatitis, primary biliary cirrhosis (PBC), sclerosing cholangitis, hemochromatosis, α1-antitrypsin deficiency, Wilson's disease, and current or past consumption of more than 20 g of alcohol daily. No patients had taken medicine which cause fatty liver, such as amiodarone, diltiazem, tamoxifen, steroids [24]. None of the patients had any clinical evidence of hepatic decompensation, such as hepatic encephalopathy, ascites, variceal bleeding, or elevated serum bilirubin level to more than twofold the upper limit of normal. Patients with heart failure or autoimmune rheumatic disease, which have been suggested to increase plasma PTX3 levels [25,26], and patients with a history of treatment with statins, which reduce plasma PTX3 levels [27], were also excluded.

### Clinical and laboratory evaluation

Body weight and height were measured with a calibrated scale after requesting the patients to remove their shoes and any heavy clothing. Whole blood from 70 NAFLD patients and 10 healthy control subjects was immediately collected into a tube containing ethylene diaminetetraacetate (EDTA), and after centrifugation at 1500 × *g *for 15 min at room temperature, the plasma was frozen, and stored at -80°C until analyzed. Serum samples were obtained from 70 NAFLD patients after an overnight fast (12 hours) to measure AST, ALT, GGT, albmin, glucose, insulin, type IV collagen 7s domain, and hyaluronic acid levels. The serum insulin levels were measured by radioimmunoassay, and the other values were measured in a conventional automated analyzer.

Insulin resistance was calculated by the modified homeostasis model assessment of insulin resistance (HOMA-IR), using the following formula: HOMA-IR = fasting insulin (μU/ml) × plasma glucose (mg/dl)/405. HOMA-IR was originally reported by Matthews, and it has since been modified [28]. This index has been shown to be closely correlated with the results of the euglycemic-hyperinsulinemic clamp method to determine insulin resistance in type 2 DM patients.

### Pentraxin3 measurements

Plasma PTX3 levels were measured with a sandwich ELISA based on a previously described method [26]. Briefly, the assay plate was coated with the F(ab')2 fragment of a monoclonal anti-human PTX3 antibody, PPMX0104 (Perseus Proteomics Inc., Tokyo, Japan) as the capture antibody and the F(ab')2 fragment of a monoclonal ant-human PTX3 antibody, PPMX0105 (Perseus Proteomics Inc., Tokyo, Japan) was conjugated with horseradish peroxidase as the detection antibody. Sample dilution (100 μl) was added to the wells. Each calibrator or plasma samples (10 μl) were added to the wells and incubated for 1 hour with shaking. The wells were aspirated and washed five times with washing buffer. PPMX0105-enzyme conjugate (100 μl) was added to the wells and incubated for 1 hour with shaking. The wells were aspirated and washed again, and substrate solution was added to each well. After 30 min of incubation, stop solution of the kit (100 μl) was added. The absorbance at 450 nm was measured with a microplate reader system. This assay system measured plasma PTX3 concentration linearly between 0.1 and 20 ng/ml.

### Determination of the visceral and subcutaneous fat areas

The abdominal fat distribution of the subjects was determined by computed tomography (CT), conducted with the subjects in the supine position in accordance with a previously described procedure [29]. The subcutaneous fat area (SFA) and intra-abdominal visceral fat area (VFA) were measured at the level of the umbilicus in terms of the CT number, by a standardized method. In brief, a region of interest was defined in the subcutaneous fat layer by tracing its contour on each scan, and the attenuation range for fat tissue was measured in terms of the CT number (in Hounsfield units).

### Pathology

The biopsy specimens of liver tissue were stained with hematoxylin-eosin, reticulin, and Masson trichrome stain, and all biopsy specimens were analyzed by one experienced pathologist blinded to the results of clinical data. Macrovesicular steatosis affecting at least 5% of the hepatocytes was observed in every case, and the cases were classified as having steatosis or steatohepatitis. In addition to steatosis, the minimal criteria for the diagnosis of steatohepatitis include the presence of lobular inflammation and either ballooning of cells or perisinusoidal/pericellular fibrosis in zone 3 of the hepatic acini [30-32]. Subjects with cirrhosis were defined as cases of NASH-associated cirrhosis according to a previously proposed clinicopathological classification [32]. The severity of fibrosis in every case was scored according to the method of Brunt [33]. The degree of steatosis was graded as follows based on the percentage of hepatocytes containing macrovesicular fat droplets: grade 0, no steatosis; grade 1, < 33% hepatocytes contain macrovesicular fat droplets; grade 2, 33% – 66% of the hepatocytes contain macrovesicular fat droplets; grade 3, > 66% of the hepatocytes contain macrovesicular fat droplets. The stages of fibrosis were expressed on the following 4-point scale, as follows: stage 0, none; stage 1, perivenular and/or perisinusoidal fibrosis in zone 3; stage 2, combined pericellular portal fibrosis; stage 3, septal/bridging fibrosis; stage 4, cirrhosis. Based on this classification, the subjects were grouped into 2 groups, a group with stages 0–2 NAFLD and a group with stages 3–4 NAFLD.

### Statistical analysis

Data were expressed as means ± SD, unless indicated otherwise. The statistical analysis was performed with SPSS 12.0 software (SPSS, Inc., Chicago, IL, USA). The *t*-test or Wilcoxon rank sum test, as appropriate, was used for univariate comparisons between patient groups. Because many of the variables were not normally distributed, the Kruskal-Wallis test was used for comparisons of more than two independent groups. The diagnostic performance of the plasma PTX3 level was assessed by analysis of receiver operating characteristic (ROC) curves. The ROC curve is a plot of sensitivity versus 1 – specificity for all possible cutoff values. The most commonly used index of accuracy is the area under the ROC curve (AUROC), with values close to 1.0 indicating high diagnostic accuracy. Calculations of correlation coefficients and linear regression analysis were used to test for associations between the variables. Multivariate analysis was performed by using a binary logistic regression analysis. P values < 0.05 were considered significant.

## Results

### Patients Characteristics

**Figure 1:**
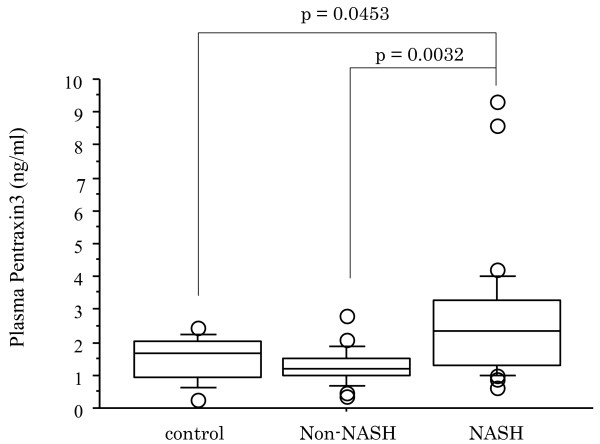
**Plasma PTX3 levels in healthy control subjects, non-NASH patients, and NASH patients**. Box plots of plasma PTX3 levels showing interquartile range (box), median (thick line), range (thin lines) and outliers (circles). The length of the box represents the interquatile range within which 50% of the values were located. Plasma PTX3 levels of healthy control subjects, non-NASH and NASH patients.

**Figure 2:**
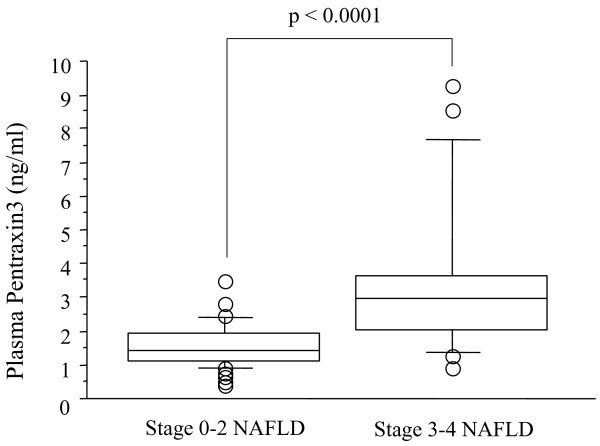
**Plasma PTX3 levels in stages 0–2 and stages 3–4 NAFLD**. Box plots of plasma PTX3 levels showing interquartile range (box), median (thick line), range (thin lines) and outliers (circles). The length of the box represents the interquatile range within which 50% of the values were located. Plasma PTX3 levels in stages 0-2 NAFLD and stages 3-4 NAFLD.

The histological findings in the liver biopsy specimens of the subjects with non-NASH (n = 28) and steatohepatitis (NASH) (n = 42) are shown in Table 1. The clinical and biochemical characteristics of the NASH patients and non-NASH patients are shown in Table 2. Marked elevation of the plasma PTX3 level was observed in the patients with NASH in comparison with the patients with non-NASH (p = 0.0021) and healthy control subjects (p = 0.0453). And there was no significance between non-NASH and control subjects in the plasma PTX3 level (p = 0.2892) (Fig. 1). Significant differences were also found in the serum AST level (p = 0.0031), ALT level (p = 0.0115), and hyaluronic acid level (p = 0.0373) between the NASH patients and those with non-NASH patients (Table 2). The clinical and biochemical characteristics of stages 0–2 NAFLD group and stages 3–4 NAFLD group are shown in Table 3. A significantly higher plasma PTX3 (p < 0.0001) was found in stages 3–4 NAFLD group in comparison with stages 0–2 NAFLD group (Fig. 2), and a markedly higher hyaluronic acid level (p = 0.0090) and type IV collagen 7s level (p = 0.0018) and markedly lower serum albumin level (p = 0.017) and platelet count (p = 0.0134) were observed between two groups (Table 3).

**Table 1:** Histopathological Findings in the NASH patients and non-NASH patients

	(NASH) (n = 42)	Non-NASH (n = 28)
Steatosis grade		
1	22	20
2	17	6
3	3	2
Inflammatory activity		
0	4	11
1	27	17
2	9	0
3	2	0
Fibrosis stage		
0	0	28
1	21	0
2	4	0
3	11	0
4	6	0

**Table 2:** Clinical and Biochemical Characteristics of the non-NASH Patients and NASH Patients

	Non-NASH patients	NASH patients	P value
Age (years)	49.0 ± 15.3	52.8 ± 13.5	0.2995
BMI (m/kg2)	27.6 ± 5.1	28.9 ± 6.0	0.3989
VFA (cm2)	128.6 ± 55.7	133.3 ± 58.8	0.8031
SFA (cm2)	196.9 ± 61.4	232.5 ± 120.9	0.3765
AST (U/ml)	35.7 ± 17.5	63.4 ± 40.7	0.0031
ALT (U/ml)	56.8 ± 31.5	89.9 ± 55.7	0.0115
FBS (mg/dl)	118.7 ± 39.9	118.2 ± 30.8	0.9586
IRI (ul/ml)	13.6 ± 10.8	14.3 ± 8.2	0.7999
HOMA-IR	3.47 ± 2.64	4.05 ± 2.54	0.4725
HDL cholesterol (mg/l)	50.3 ± 12.6	49.3 ± 11.4	0.7463
LDL cholesterol (mg/l)	133.7 ± 27.0	128.6 ± 39.3	0.5928
Triglyceride (mg/l)	159.0 ± 48.9	162.8 ± 81.9	0.8502
Albumin	4.51 ± 0.28	4.45 ± 0.51	0.6175
Platelet count	25.8 ± 5.7	23.2 ± 8.0	0.1816
Hyaluronic acid (ng/dl)	21.6 ± 15.1	50.1 ± 50.2	0.0373
Type IV collagen 7s (ng/dl)	4.41 ± 0.98	5.14 ± 1.31	0.0611

Data are expressed as means ± SD. VFA: visceral fat area, SFA: subcutaneous fat area, AST: aspartate aminotransferase, ALT: alanine aminotransferase, FBS: fasting blood sugar, IRI: immunoreactive insulin, HOMA-IR: homeostasis model assessment of insulin resistance.

**Table 3:** Clinical and Biochemical Characteristics of Stages 0–2 and Stages 3–4 NAFLD

	Stages 0–2 NAFLD	Stages 3–4 NAFLD	P value
Age (years)	51.5 ± 13.4	52.8 ± 15.1	0.7564
BMI (m/kg2)	28.1 ± 4.9	30.2 ± 6.7	0.2133
VFA (cm2)	132.1 ± 63.7	135.7 ± 33.4	0.8716
SFA (cm2)	216.9 ± 95.3	256.8 ± 108.2	0.0626
AST (U/ml)	54.6 ± 41.0	58.1 ± 26.9	0.7526
ALT (U/ml)	80.3 ± 49.3	82.4 ± 59.5	0.8903
FBS (mg/dl)	122.9 ± 39.9	112.3 ± 17.8	0.3598
IRI (ul/ml)	13.0 ± 9.06	18.3 ± 9.10	0.0762
HOMA-IR	3.56 ± 2.59	5.16 ± 2.43	0.0677
HDL cholesterol (mg/l)	50.1 ± 12.5	47.8 ± 10.2	0.5277
LDL cholesterol (mg/l)	130.6 ± 33.3	129.8 ± 41.0	0.9430
Albumin (mg/l)	4.55 ± 0.27	4.23 ± 0.66	0.0170
Platelet count (ng/ml)	25.8 ± 6.6	20.0 ± 8.7	0.0134
Hyaluronic acid (ng/dl)	32.7 ± 27.8	72.4 ± 68.1	0.0090
Type IV collagen 7s (ng/dl)	4.65 ± 0.93	5.94 ± 1.53	0.0018

Data are expressed as means ± SD. VFA: visceral fat area, SFA: subcutaneous fat area, AST: aspartate aminotransferase, ALT: alanine aminotransferase, FBS: fasting blood sugar, IRI: immunoreactive insulin, HOMA-IR: homeostasis model assessment of insulin resistance.

### Hepatic fibrosis and plasma PTX3 levels

**Figure 3:**
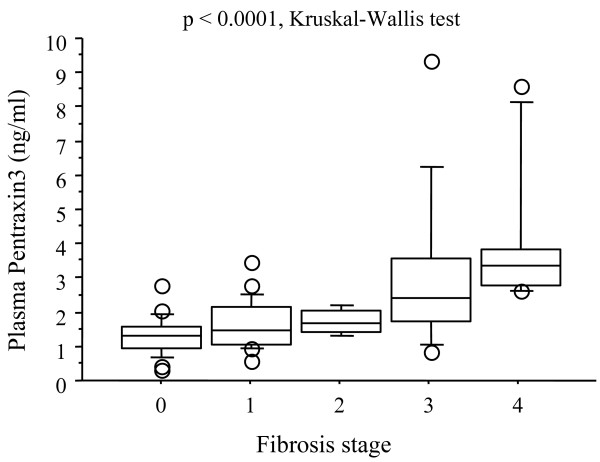
**Relation between Plasma PTX3 levels and fibrosis stage**. Comparison between plasma PTX3 levels in various stages of fibrosis. Box plots of plasma PTX3 levels showing interquartile range (box), median (thick line), range (thin lines) and outliers (circles). The length of the box represents the interquatile range within which 50% of the values were located. A steady stepwise increase in serum high-sensitivity CRP levels was observed with increasing severity of hepatic fibrosis (p = 0.0049).

Since the plasma PTX3 level was significantly elevated in the NASH patients in comparison with the non-NASH patients, and in stages 3–4 NAFLD group in comparison with stages 0–2 NAFLD group, we investigated the relationship between stage of fibrosis and the plasma PTX3 levels in the NAFLD patients. Analysis of the plasma PTX3 levels of the NAFLD patients in relation to the histological stage of fibrosis revealed stepwise increases in the plasma PTX3 levels as the stages of hepatic fibrosis increased (p < 0.0001, Kruskal-Wallis test) (Fig. 3), and the differences between stage 0 and stage 3, stage 0 and stage 4, stage 1 and stage 3, stage 1 and stage 4, and stage 2 and stage 4 were significant (p = 0.0009, p < 0.0001, p = 0.0136, p = 0.0001, p = 0.0465, respectively).

### Relation between plasma PTX3 and grade of hepatic steatosis or grade of necroinflammation

Analysis of the plasma PTX3 levels in relation to the histological grade of steatosis or grade of necroinflammation showed no relation between the plasma PTX3 levels and either of the two other parameters (p = 0.1554, p = 0.1745, respectively by Kruskal-Wallis test).

### Receiver operating characteristic (ROC) curves for differentiating between NASH and non-NASH based on the plasma PTX3 level

**Figure 4:**
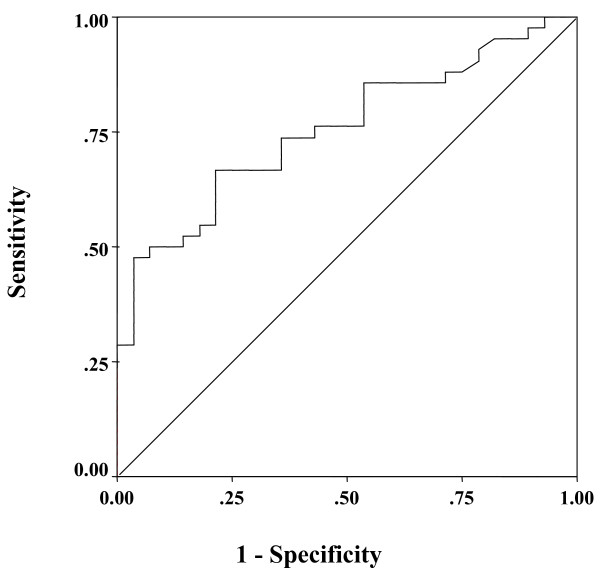
**ROC curve for differentiating steatosis and NASH according to the plasma PTX3 level**. ROC curve for differentiating between steatosis and NASH on the basis of the plasma PTX3 level (area under the curve = 0.719).

To detect NASH compared with non-NASH, the area under the curve for plasma PTX3 was 0.755 by ROC analysis (Fig. 4). The best cutoff value for diagnosis of NASH was also investigated by ROC analysis, and sensitivity, specificity, positive predictive value (PPV), and negative predictive value (NPV) were calculated. The results showed that 1.61 ng/ml was the optimal plasma PTX3 cutoff value for diagnosis of NASH, and its sensitivity, specificity, PPV, and NPV were 66.7%, 78.6%, 82.4%, and 61.1%, respectively.

### ROC curves for differentiating between stages 0–2 NAFLD and stages 3–4 NAFLD based on the plasma PTX3 level

**Figure 5:**
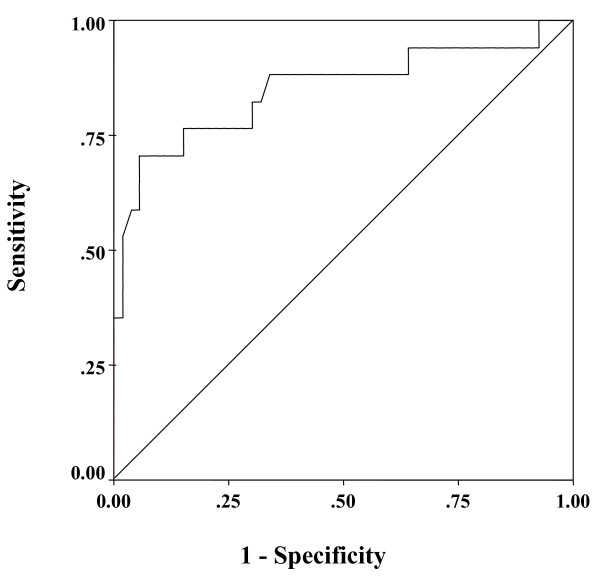
**ROC curve for differentiating between advanced NAFLD and non-advanced NAFLD on the basis of the plasma PTX3 level**. ROC curve for differentiating between non-advanced NAFLD and advanced NAFLD on the basis of the plasma PTX3 level (area under the curve = 0.849).

To detect stages 3–4 NAFLD compared with stages 0–2 NAFLD, the area under the curves for plasma PTX3 were 0.850 by ROC analysis (Fig. 5). The results of the analysis showed 2.45 ng/ml to be the optimal plasma PTX3 cutoff value for diagnosis of advanced NAFLD, and its sensitivity, specificity, PPV, and NPV were 70.6%, 94.3%, 80.8%, and 90.9%, respectively.

### Multiple regression analysis for demographic factors in the NASH patients

We performed the multiple logistic regression analysis by using the plasma PTX3 levels, AST, ALT, type IV collagen 7s domain, hyaluronic acid, which were significantly elevated factors in NASH patients than in non-NASH patients by the univariate analysis. Plasma PTX3 level was still significant in the NASH patients as compared with that in non-NASH patients by multiple logistic regression analysis (Table 4).

**Table 4:** Multiple Logistic Regression Analysis of Factors Associated with NASH Compared to non-NASH.

Factor	Odds ration	95% CI	p Value
plasma PTX3 level (ng/ml)	0.254	0.059 – 0.984	0.0488
AST (U/ml)	0.984	0.910 – 1.064	0.692
ALT (U/ml)	1.008	0.954 – 1.065	0.7699
Type IV collagen 7s domain (ng/ml)	1.128	0.407 – 3.124	0.8171
hyaluronic acid (ng/ml)	0.965	0.908 – 1.025	0.2499

Note: Intercept = -19.493, R2 for entire model = 0.310

### Correlation between plasma PTX levels and serum CRP levels in the NAFLD patients

**Figure 6:**
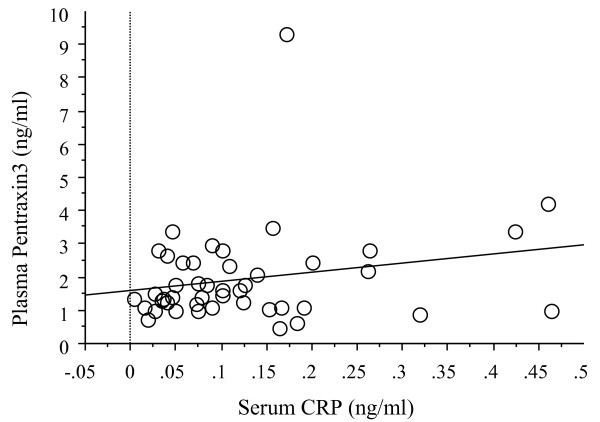
**Correlation between the plasma PTX3 levels and the serum CRP levels**. No correlation was found between plasma PTX3 levels and serum CRP levels (r = 0.220, p = 0.1431)

We compared the plasma PTX3 levels and serum CRP levels in 70 NAFLD patients. No correlation was found between plasma PTX3 levels and serum CRP levels (r = 0.220, p = 0.1431) (Fig. 6).

## Discussion

The results of this study demonstrated markedly higher plasma PTX3 levels in the NASH patients in a comparison with the non-NASH patients, and in stages 3–4 NAFLD as compared with stages 0–2 NAFLD.

A growing body of evidence has recently been collected that supports the notion that NAFLD is a feature of the metabolic syndrome. NAFLD sometimes progresses to cirrhosis, it is considered to be the most common form of chronic liver disease in obese patients. Because there are few effective treatments for NAFLD, the identification of important risk factors for disease progression should provide valuable information in regard to both risk stratification and development of risk-reduction strategies [34].

C-reactive protein (CRP) is one of the major acute-phase proteins and a marker of systemic inflammation. Some recent reports have suggested that hs-CRP may be an independent risk factor for progression of NAFLD [35-37], and we have reported the clinical usefulness of the measurement of hs-CRP for diagnosis of NASH [10]. PTX3 is a prototypic member of the long pentraxin family and a newly discovered marker of the acute phase inflammatory response. PTX3 is structurally related to, but distinct from classic members of the short pentraxin family, including from serum amyloid protein and CRP. The short pentraxins CRP and serum amyloid protein are reported to be produced by the liver as a systemic response to local inflammation, whereas expression of the long pentraxin PTX3 is rapidly induced in damaged tissue and may more directly reflect the tissue inflammatory response and its expression pattern is more tissue specific [21,38]. The gene coding PTX3 was originally described as an interleukin (IL)-1β-inducible gene in endothelial cells [14] and a tumor necrosis factor (TNF)-α inducible gene in fibroblasts [15]. PTX3 is produced by a variety of cell types, including monocytes/macrophages and endothelial cells in response to pro-inflammatory stimuli, such as TNF-α, IL-1β, and lipopolysaccharide (LPS) [39], all of which are reported to be essential factors in the pathogenesis and progression of NASH [40-42]. Patients with NAFLD are well known to have metabolic syndrome, and increasing number of metabolic syndrome risk factors is associated with increasing CRP [43,44]. Furthermore, metabolic syndrome in patients with NAFLD was shown to be associated with greater histologic severity [45]. However Inoue K et al. reported that PTX3 is an independent factor from metabolic risk factors, unlike CRP [46]. And previous report [21] and this study showed no correlation between plasma concentrations of PTX3 and CRP. Because of the similarities and differences between PTX3 and CRP, it is important to assess the usefulness of PTX3 as a novel diagnostic tool for NASH.

We therefore investigated the relationship between plasma PTX3 levels and several stages of NAFLD, including NASH. The results demonstrated that plasma PTX3 levels can be used to reliably differentiate NASH patients from non-NASH patients. A more detailed study revealed that plasma PTX3 levels can be used to differentiate between stages 3–4 NAFLD and stages 0–2 NAFLD, and that higher plasma PTX3 levels in NAFLD patients are associated with severe stages of hepatic fibrosis. Plasma PTX3 levels in healthy volunteers showed around 1.5 ng/ml, and plasma PTX3 levels in NASH patients were around 2.6 ng/ml. Furthermore in patients with stage 3–4 NAFLD patients was further elevated (around 3.8 ng/ml). Thus, we showed that the baseline plasma PTX3 level is not only a strong clinical marker of NASH, but also of the severity of liver fibrosis in NAFLD patients. PTX3 is reported to bind to apoptotic cells inhibiting their recognition by dendritic cells [47], and there is evidence for a regulatory role of PTX3 in noninfectious inflammatory reactions [48].

Measurement of plasma PTX3 levels is likely to be useful for targeted therapies for primary prevention of cardiovascular disease [49]. Similarly, plasma PTX3 may be also useful for targeted therapy against fibrosis in NASH patients, because in our study, the plasma PTX3 level was found to be more significantly elevated in the advanced stages of NAFLD patient.

## Conclusion

This is the first study to demonstrate a consistent and profound elevation of plasma PTX3 levels in NASH patients whose diagnosis was based on results of liver biopsy, the gold standard for the diagnosis of NASH. Plasma PTX3 measurements may be noninvasive and appear to show promise as a means to differentiate NASH from non-NASH patients, and as a clinical marker of the severity of liver fibrosis in NASH patients.

## Abbreviations

PTX3: pentraxin3; CRP: C-reactive protein; VFA: visceral fat area; BMI: body mass index; SFA: subcutaneous fat area; HOMA-IR: homeostasis model assessment for insulin resistance; IL: interleukin.

## Competing interests

The authors declare that they have no competing interests.

## Authors' contributions

MY and TU performed the literature review, collected the clinical data, and drafted the manuscript, with contributions from MS, HA, and TK. SK and YN organized the field survey for data collection. HE, HM, HI, TK, KF, KY, HT, HK, MI, NK, KK and SS collected the clinical data. SM analyzed the liver pathology. AN was responsible for the design of the study. All authors read and approved the final manuscript.

## Pre-publication history

The pre-publication history for this paper can be accessed here:


